# Can physics review catch prescription and PTV margin errors in fully automated radiotherapy plans? A hazard study

**DOI:** 10.1002/acm2.70759

**Published:** 2026-09-28

**Authors:** Laurence E. Court, Raphael Douglas, Parmeswaran Diagaradjane, Vindu W. Kathriarachchi, Shiquin S, Ergys Subashi, Yao Zhao, Lifei Zhang, Tucker Netherton

**Affiliations:** ^1^ Department of Radiation Physics University of Texas MD Anderson Cancer Center Houston Texas USA

**Keywords:** automated planning, radiotherapy planning, risk management

## Abstract

**Purpose:**

End‐to‐end AI‐based automated contouring and radiotherapy planning systems promise substantial gains in efficiency and consistency but also introduce new safety risks, including user input errors that occur early in the workflow. This study evaluates the ability of standard physics plan review to detect user errors in prescription and planning target volume (PTV) margin entry in a fully automated planning workflow.

**Methods:**

A prototype of the Radiation Planning Assistant (RPA) was used to generate fully automated prostate cancer treatment plans for 20 patients. Five cases intentionally included incorrect prescriptions or non‐standard PTV margins. Five clinical medical physicists independently performed physics plan reviews in a commercial treatment planning system, assuming a standard clinical workflow and without access to user guides or service request forms. Reviews were conducted in two phases, with and without margin information embedded in structure names. A third phase evaluated whether adding reference expansion structures improved margin error detection.

**Results:**

Incorrect prescriptions were detected in 80% of cases, with no false‐positive identification of correct prescriptions. In contrast, detection of incorrect PTV margins was highly inconsistent. Two physicists did not evaluate margins and missed all margin errors. Three physicists identified all intentional margin errors but demonstrated poor specificity, frequently flagging correct cases as incorrect (specificity range: 13%–63%). Including margin information in structure names and adding reference expansion structures did not improve specificity or overall detection performance.

**Conclusions:**

Physics plan review was unreliable for detecting incorrect PTV margins in a fully automated planning workflow, even when additional cues were provided. Although based on a limited dataset, these findings indicate that traditional plan review processes alone are insufficient to mitigate this failure mode. Alternative risk‐reduction strategies—such as timely workflow‐embedded alerts, targeted checklists, automated verification tools, and careful expectation setting—are likely required to improve safety in AI‐driven radiotherapy planning systems

## 1 INTRODUCTION

End‐to‐end AI‐based automated contouring and radiotherapy planning are expected to become commonplace in the next few years, with early clinical applications and feasibility already reported in the literature.^1^, ^2^, ^3^ This advance will bring gains in efficiency, quality and consistency. By helping clinical teams scale their efforts to treat more patients, automation may help improve equitable access to radiotherapy across the world. However, automation will also change the risk profile of radiotherapy planning, potentially introducing new risks like automation bias, off‐label use, software error, and workflow differences.^4^ Several studies have shown a major failure mode will still be user error, even when most tasks are fully automated.^4^, ^5^ Thus, these new systems should be designed to minimize likelihood of user error and, for situations when user errors do occur, to maximize the likelihood of error detection.

One example of a software tool that is being built to support automated radiotherapy planning is the Radiation Planning Assistant (RPA).^6^, ^7^ The RPA has been designed specifically to support clinics in low‐ and middle‐income countries. A prototype of a future version of the RPA (i.e., not yet clinical) has been developed to support full end‐to‐end contouring and planning for prostate and cervical cancers. In that system, the user must enter the prescription, treatment targets (prostate, pelvic nodes, etc.), and planning target volume (PTV) margins.^7^ Although contouring and treatment plan generation are automated, user error remains a potential failure mode. The RPA user interface was designed following principles outlined in IEC 62366^8^ and has previously undergone extensive usability testing.^9^

Given the potential impact of user input errors, this study focuses on two specific failure modes in end‐to‐end planning using the RPA—prescription entry errors and PTV margin entry errors—and evaluates the likelihood that such errors would be detected during the physics plan‐check process. These are both error modes that were identified as high‐risk failure modes by TG275.^10^, ^11^ In addition, inadequate manual review of fully automated plans has been identified as a high scoring failure mode.^12^ Although the user interface limits the range of values that can be entered, there is still a risk that the user will enter incorrect values. Although the RPA, like most commercial treatment planning systems, generates a written report describing the plan, there is no guarantee that users will review it. Similarly, warnings about possible failure modes should be included in the user guides, but many users will not have read these. Therefore, approaches that increase the likelihood of identifying prescription or margin errors without requiring review of written reports or guides are desirable.

Specifically, this work uses hazard testing to assess whether physics plan review is likely to catch user input errors that happened earlier in the planning workflow. Although the RPA is used as a case study, the results are applicable to the vendor solutions that we can expect in the coming years.

## 2 MATERIALS AND METHODS

### 2.1 The RPA user interface

Figure 1 shows the user interface for the Service Request in a prototype version of the RPA. This form is completed by any clinical user and then approved by a user with radiation oncologist's rights. All numerical entry fields include permissible ranges, selected to provide sufficient flexibility while reducing the risk of incorrect (unreasonable or dangerous) data entry (following standard usability practices^8^). Different parts of the Service Request become active depending on earlier data entry—for example, the pelvic node margin selection becomes active when dose is prescribed to the pelvic nodes. In addition to completing and approving the Service Request, the user must upload a CT (see Court et al.^7^ for more details). It then automatically contours the necessary structures and creates a VMAT plan. All contours and plans in this study were created by the RPA.

**FIGURE 1:**
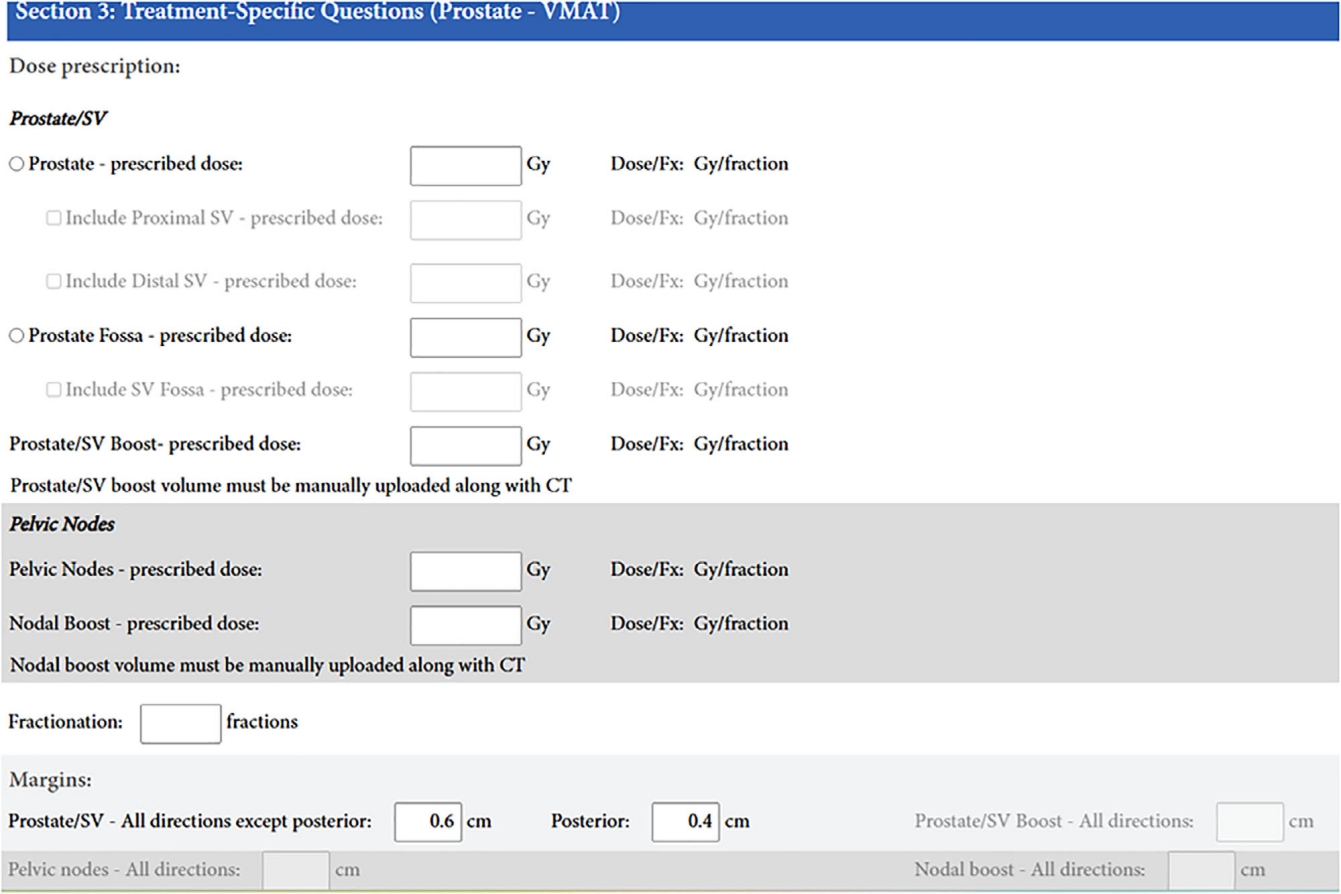
Screenshot of the service request form for fully automated contouring and planning of prostate cancer cases in the radiation planning assistant. The user selects treatment targets, radiation prescription (total dose, fractionation), and PTV margins.

### 2.2 Planning directive

The standard planning directive used in this study is given in Table 1 (PTV prescriptions), Table 2 (organs at risk dose constraints), and Table 3 (PTV margins).

**TABLE 1:** PTV prescriptions (following directive planning directive).

Planning target volume	Dose to PTV
Prostate	Report dose to PTVp_6000
	D50% = 60 Gy ± 1% (median)
	D98% ≥ 57.0 Gy (95% prescribed dose)
	D2% ≤ 64.2 Gy (107% prescribed dose)
	Aim for D2% ≤ 63.0 Gy (105%)
Seminal vesicle	D50%≥ 47.0 Gy
	D98%≥ 44.65 Gy (95%)
Pelvic node PTV	D50% ≥ 47 Gy
	D98% ≥ 44.65 Gy (95%)
	D2%≤ 50.3 Gy (107%)

**TABLE 2:** Organ at risk dose constraints (following directive planning directive).

	Dose volume constraints		
		Maximum volume (% or cc)	
Organ at risk	Dose (Gy)	Mandatory	Optimal
Rectum	24	80%	70%
	32	65%	51%
	40	50%	38%
	48	35%	27%
	52	30%	_
	56	15%	_
	60	3%	1%
Bladder	40	50%	_
	48	25%	_
	60	15%	5%
Femoral heads	40	50%	5%
Bowel	40	70cc	17cc
	48	6cc	0.5cc
	52	0cc	_
Penile bulb	22	_	<50%
	48	_	10%

**TABLE 3:** PTV margins (following directive planning directive).

PTV	Margin
Prostate and SV	4 mm posterior, 6 mm all other directions
Pelvic nodes	5 mm all directions

#### 2.2.1 Treatment targets

Entire prostate, entire SV, pelvic nodes (depends on the patient characteristics).

#### 2.2.2 Dose objectives and constraints

#### 2.2.3 PTV margins

### 2.3 Plan dataset

Twenty CT scans from previously treated prostate cancer patients were identified. This number was chosen as a practical limit, based on the amount of time that we expected the participants to be able to dedicate to this study. Of these 20 cases, 10 were typical cases from our clinic, and the remaining 10 were intentionally difficult cases from the Cancer Imaging Archive (TCIA) Prostate Anatomical Edge Cases database.^13^ Although contouring and planning issues were not the focus of this study, these difficult cases were included to try to add additional challenges to the study, with a range of concerns for the plan reviewers to find across the patients.

Treatment targets, prescribed dose and PTV margins were entered into the RPA Service Request form following Table 4, and automated RPA plans generated. Patients 4, 10,11, 13, and 17 are hazard cases where incorrect data were intentionally entered. As highlighted in the table, the hazard cases involve a non‐standard prescription or PTV margin. For patients 1–10, the PTVs were labeled using the standard RPA process. That is, PTV****cGy, where **** is the prescribed dose in cGy. For patients 11–20, PTVs included text about the treatment margin. For PTVs generated using the standard margins in the planning directive (Section 2.2), the PTVs were labeled PTV****cGy_StandardMargin. For PTVs generated using a different margin, the PTVs were labeled PTV****cGy_NonStandardMargin.

**TABLE 4:** Prescriptions and PTV margins entered into the RPA service request form.

Patient #	Prostate	Proximal seminal vesicles	Distal seminal vesicles	Pelvic lymph nodes	PTV margin	Structure labeling
1	60	–	–	–	Following directive	Standard
2	60	–	–	–	Following directive	Standard
3	60	–	–	–	Following directive	Standard
4	**66**	47	47	–	**3 mm isotropic**	Standard
5	60	47	47	–	Following directive	Standard
6	60	–	47	–	Following directive	Standard
7	60	–	47	47	Following directive	Standard
8	60	47	47	47	Following directive	Standard
9	60	47	47	47	Following directive	Standard
10	60	47	–	47	**3 mm isotropic**	Standard
11	60	–	–	–	**3 mm isotropic**	Include margin text
12	60	–	–	–	Following directive	Include margin text
13	**66**	–	–	–	Following directive	Include margin text
14	60	47	47	–	Following directive	Include margin text
15	60	47	47	–	Following directive	Include margin text
16	60	47	47	–	Following directive	Include margin text
17	60	47	–	47	**3 mm isotropic**	Include margin text
18	60	47	–	47	Following directive	Include margin text
19	60	47	47	47	Following directive	Include margin text
20	60	47	–	47	Following directive	Include margin text

*Note*: Hazard cases are highlighted.

### 2.4 Physics plan review

The design of this experiment assumes that users often do not read user guides (so these are not provided), and that other documentation may not be available to the plan reviewer. This assumption is supported by both our institutional experience and published literature. In the case of the RPA, we are assuming that the reviewer may not review the Service Request form for consistency/correctness, and they just review the treatment plan in their treatment planning system.

Five clinical physicists were given the following instructions (the actual instruction sheet is given in Appendix A):

- The RPA has been used to automatically generate contours and VMAT plans to treat prostate cancer following a standard directive (see Section 2.2). In this process, the plan details are entered manually, and the RPA then creates all contours and the plan automatically. These automatically generated contours/plans have been imported into the Raystation treatment planning system.
- Please perform a physics review of each patient's plan in Raystation:
  ∘ Assume we are following a standard workflow where each patient's plan has already undergone physician review.
  ∘ The contouring/planning Standard Operating Procedure (SOP) is provided (in the planning directive).
  ∘ Individual patient prescriptions and treatment targets are provided (in the standard planning directive).
  ∘ Tasks that would happen after the treatment planning system (i.e., data transfer to Oncology Information System [OIS]) are not reviewed in this study.
  ∘ Feel free to make changes to display settings (e.g., isodose levels), but do NOT save your work as others will review the same patient plan.
  ∘ Any findings should be entered into the review sheet.
- After reviewing each patient, please do not go back.
- Please do not discuss your findings with colleagues.
- Please review in two phases:
  ∘ Phase 1: Please review patients 1–10.
  ∘ Phase 2: Please review patients 11–20.
  ∘ Phase 3: Please review patients 1–10 (new reference structure included).

The third phase was added, based on the result of phase 1 and 2. In that phase, reference structures were added to patients 1–10, and the physicists were asked to re‐review these plans. The reference structures were a 5 mm isotropic expansion of the CTVs, and labeled following CTV****cGy_expanded 5 mm, where **** is the prescribed dose in cGy. Expansions were performed in RayStation, independent of the RPA's expansion engine (which uses Eclipse). This design tested if an independent secondary expansion could serve as a visual aid for detecting errors, despite inherent inter‐system variability.

No instructions were given to the physicists regarding the use of standardized tools or checklists, as they were expected to follow their routine practices. This was done to try to avoid identifying the target of the study (i.e., margin errors).

## 3 RESULTS

When assessed on the user‐generated PTV, the plans met the required dose objectives. For plans generated with incorrect PTV expansions, target coverage objectives were satisfied for the PTVs used during planning but not for the protocol‐defined PTVs. Although the D98% objectives of 57 Gy, 44.65 Gy, and 44.65 Gy were achieved for the prostate, seminal vesicle, and pelvic nodal planning PTVs, respectively, evaluation using the protocol PTVs demonstrated reduced coverage. Median D98% values decreased from 59.0 Gy to 56.2 Gy for the prostate, from 46.2 Gy to 43.6 Gy for the seminal vesicles, and from 44.9 Gy to 41.8 Gy for the pelvic nodes.

Five medical physicists reviewed all plans. These physicists had a range of clinical experience, from 3 months to 10 years (median: 5 years).

Four of the five physicists identified the incorrect prescription (*n* = 1) in phase 1. Three of the 5 physicists identified the incorrect prescriptions (*n* = 2) in phase 2. There were no instances in which correct prescriptions were flagged as incorrect.

Detection of incorrect margins was extremely varied. Two of the physicists did not comment on PTV margins for any case and missed all contouring errors. Three of the physicists identified all intentional contouring errors in both phases of the experiment (*n* = 2 for each phase). However, they also identified contouring errors for many cases where there were no contouring errors. Overall specificity for those three physicists was 13%, 31%, and 63%, and was poor for both phases of the experiment (13%/13% for one physicist for each phase, 50%/75% for one physicist, and 13%/50% for the third). Years of experience were not seen to affect whether the physicist detected or didn't detect the margin errors. The addition of the margin reference structure was also found to be unhelpful, with no improvements found in specificity. One physicist who had not previously identified any PTV margin issues began flagging such cases in the third phase after the issue of incorrect margins had been brought to their attention. In both phases, the reviewers also identified various other issues of concern. These included comments about contour quality (targets and normal tissues), comments about not meeting dose constraints (targets and normal tissues), not contouring fiducial markers, no density override of hip prosthesis, comments about anisotropic dose grids.

## 4 DISCUSSION

The reviewers were able to identify prescriptions reasonably well, with 80% of incorrect prescriptions identified overall. Specificity was also high, with no incorrect flagging of correct prescriptions. Overall, this is higher than generally reported for error detection in physics plan review.^11^, ^14^, ^15^

In this study, manual review was not an effective means of detecting PTV margin expansion errors. Sixty percent of the reviewers (3/5) included margin review in their checks—in line with the TG275 survey. The three physicists who checked the margins incorrectly flagged the majority of correct cases (as well as identifying the incorrect cases). The inclusion of non‐standard margin descriptors in the PTV naming did not influence outcomes; therefore, this strategy does not appear to be effective. When asked how they assessed the treatment margins, two responded that they did this manually, using the measuring tool available in the treatment planning system, and one responded that they recreated the expansions in the treatment planning system. Overall, they indicated that manual measurement is difficult because of the 3D nature of margins, and the small size of some structures. These results are in line with TG 275, who suggest that it is often very challenging for physicists to effectively review PTV margins,^11^ and suggest that automated verification tools may be useful. Checkers did not seem to pay much attention to the naming conventions, possibly because they did not expect to find margin information in the PTV name. Although it could be argued that drawing attention of the reviewers to the naming would help with this, we chose not to do this here because (1) that would have drawn the reviewers attention to expected error modes, and (2) our experience is that many people do not read the user documentation and so may not be aware of this.

Some reviewers did attempt to reproduce the margin expansions in their treatment planning system (RayStation), but this resulted in significant false positives. Comparisons of margin expansions generated by different software packages can be difficult to interpret because the resulting structures may differ slightly due to processing differences. In workflows such as the RPA, where the automated tool is separate from the final treatment planning system, reproducing the expansion or creating a reference expansion may therefore lead reviewers to incorrectly flag cases. These differences between treatment planning systems are, perhaps, not a surprise, and are reported elsewhere.^16^, ^17^ Based on this limited literature, agreement of < 1 mm seems like a reasonable expectation.^18^

Overall, this means that, for automated tools like the RPA where users manually enter PTV margins, we cannot rely on the physics review process to reliably flag the use of incorrect margins, and additional measures are needed. This finding is in line with work of other authors,^11^, ^12^ although we believe this is the first time that it has been experimentally demonstrated. A focus group discussion held between the project participants led to the following proposals, in approximate order of likely effectiveness. Note, these are based on discussion of the study participants and the fact that the solutions attempted here were not sufficiently successful. Additional work would be needed to determine the actual effectiveness of these proposals.

- **Institution‐specific default margins**. The use of predefined default margins may improve safety by reducing opportunities for incorrect margin entry. As a mitigation strategy, the RPA user interface is being revised to support institution‐specific default margins, thereby reducing the need for manual margin entry.
- **Embedded alerts**. Include alerts to users when they change margins from default values, stating that margin errors are difficult to catch and any changes should be carefully considered. This is supported by published evidence that early warnings can be effective, although they must be carefully designed to timely and actionable and avoid ‘alarm fatigue.^19^, ^20^’ In the case of the RPA, this means including alerts when the user is completing the Service Request so that they can immediately remedy any unintentional data entry. This change is being made to the RPA user interface.
- **Check‐list**. Create a list of high‐risk items to support workflow controls and plan‐review checklists. This recommendation is consistent with prior work supporting the value of checklists for the safe introduction of AI‐based planning.^11^, ^21^, ^22^
- **Automated verification**. Provide automated verification tools for the user to use in their own treatment planning system (as suggested by TG27511, especially for PTV margin review). Substantial evidence indicates that automation can improve the effectiveness of the plan‐checking process.^23^, ^24^
- **Expectation setting**. Carefully set expectations during user training. Mismatched user expectations are known to be an important source of use errors.^25^, ^26^ Here, users should be reminded that user data entry error is a potentially high‐risk failure mode, that manual evaluation of margins is difficult, and different planning systems create margins slightly differently—thus helping the user appreciate the value of plan checks and the expected variability in that process. User training may have limited long‐term impact because it only reaches users who attend the training and because retention of detailed information declines over time. Generally, modifying user interface or device workflow design is considered more effective than training or labeling.^25^
- **User guide**. Include warnings in the user guides. Although some users will appreciate these warnings, it is known that users frequently do not read instruction manuals in their entirety, and engagement with warnings in such documents is highly variable.^27^ Providing written safety information is generally not considered to be as effective as other approaches such as ‘inherent safety by design’ or alerts that are built into the tool.^25^

Limitations to this study include the limited number of reviewers (*n* = 5), the fact that they are all from the same institution, as well as the limited number of hazard cases (five cases out of 20 total), and the fact that we only considered prostate plans. However, because the results were negative (i.e., review was not effective in detecting user error), we believe the overall conclusions are unlikely to change with increased testing. The retrospective nature of this study may also be a limitation, although the plan reviewers did appear to review the plans in detail. This study focused on the physics plan review process, and did not investigate other important review steps, such as review by radiation oncologists and therapists.

## 5 CONCLUSION

We conducted a hazard study to investigate the detectability of user errors in a fully automated end‐to‐end contouring/planning process. Specifically, we examined detectability of incorrect PTV margin entries inconsistent with their usual clinical practice. Although our dataset was limited in size, we found that inclusion of margin information in the structure name did not improve detectability, and plan reviewers often incorrectly flagged cases that did not have an error. No approach was found to improve this, so these failure modes should be mitigated with alternative approaches, especially those which reduce likelihood of user error.

## AUTHOR CONTRIBUTIONS

All authors contributed to the implementation of the research, data collection, the analysis of the results, and writing the manuscript.

## CONFLICT OF INTEREST STATEMENT

The Radiation Planning Assistant project has been funded by the NI/NCI, Varian Medical Systems, Cancer Prevention Research Institute of Texas, Wellcome Trust and the University of Texas MD Anderson Cancer Center.

## ETHICS STATEMENT

The use of patient data in this retrospective study was approved the IRB of MD Anderson Cancer Center (PA16‐0379).

## Data Availability

All experimental data are included in the paper text.
